# Clinical Correlates of FDG PET and DaT Factors in Heterogenous Clinical Sample

**DOI:** 10.1002/alz70856_107272

**Published:** 2026-01-08

**Authors:** Jessica May Pommy, Manisha Sawhney, Soumya Bouchachi, Douglas W. Scharre

**Affiliations:** ^1^ Ohio State University Wexner Medical Center, Columbus, OH, USA

## Abstract

**Background:**

There is considerable variability among patients with Lewy body disease (LBD) and Alzheimer's disease (AD). Although neuroimaging and clinical assessments can help distinguish between the two conditions, significant overlap still exists. Further research is required to better understand the neurobiological factors linked to biomarkers, cognitive function, and clinical symptoms in these diseases.

**Method:**

As part of a larger ongoing study, participants diagnosed with Alzheimer's disease (AD) or Lewy body disease (LBD) were recruited from movement and memory disorders clinics. They underwent cognitive testing (SAGE, MMSE), neuroimaging (including FDG PET, amyloid PET, and DaTscan), motor assessment (MDS‐UPDRS, Berg Balance Scale), and completed symptom questionnaires. Collateral completed an ADL rating form as well. Clinical ratings from FDG PET and DaTscan were analyzed using principal components analysis (PCA). Neuroimaging factor scores were examined in relation to amyloid positivity and assessment measures mentioned using nonparametric statistical methods. Present analyses were limited to participants (mean age of 69 years, range 47‐ 81 years) with complete DaTscan, FDG PET and amyloid imaging.

**Result:**

The PCA revealed five factors based, including: 1) subcortical FDG, 2) fronto‐parietal FDG, 3) bilateral basal ganglia (BG) DaTscan, 4) bilateral occipital FDG, and 5) right frontal FDG. Amyloid positivity status was related to fronto‐parietal FDG and bilateral BG DaTscan (*p* <.001, *p* = .019, respectively). Correlational analyses revealed the fronto‐parietal FDG factor was significantly related to scores on SAGE, MMSE, and ADLs (*p*‐values < .001). SAGE score was also negatively correlated with bilateral BG DaTscan (*p* = .012). Follow up analyses within the DaTscan positive group revealed somatic perceptions (*p* = .01) and fluctuations in alertness (*p* = .047) were associated with subcortical FDG, while motor functioning was associated with frontoparietal FDG (*p* = .041) and BG DaTscan (*p* = .033).

**Conclusion:**

Overall, our approach identified distinct FDG and DaTscan factors using clinical ratings when assessed within the same statistical model. These factors were differentially related to amyloid positivity, cognition, functioning, and select symptom measures. This approach may hold utility in clinical datasets which are often comprised of more complex patients and may rely on clinical reads of PET and DaTscan.

